# Tailoring Copolymer Properties by Gradual Changes in the Distribution of the Chains Composition Using Semicontinuous Emulsion Polymerization

**DOI:** 10.3390/polym9020072

**Published:** 2017-02-22

**Authors:** Carlos Federico Jasso-Gastinel, Alvaro H. Arnez-Prado, Francisco José Aranda-García, Luis Octavio Sahagún-Aguilar, Fernando A. López-Dellamary Toral, María Elena Hernández-Hernández, Luis Javier González-Ortiz

**Affiliations:** 1 Chemical Engineering Department, Universidad de Guadalajara, 44100 Guadalajara, Mexico; alvaro.arnez@gmail.com (A.H.A.-P.); iq.pacoaranda@gmail.com (F.J.A.-G.); luisoctaviosahagun@hotmail.com (L.O.S.-A.); maelena.hernandez@gmail.com (M.E.H.-H.); 2 Chemistry Department, Universidad de Guadalajara, 44100 Guadalajara, Mexico; ferdellam@gmail.com (F.A.L.-D.T.); ljglez@yahoo.com.mx (L.J.G.-O.)

**Keywords:** copolymer, free radical, mechanical properties, semicontinuous polymerization, composition distribution, gradual composition changes, synergic performance

## Abstract

To design the properties of a copolymer using free radical polymerization, a semicontinuous process can be applied to vary the instantaneous copolymer composition along the conversion searching for a specific composition spectrum of copolymer chains, which can be termed as weight composition distribution (WCD) of copolymer chains. Here, the styrene-*n*-butyl acrylate (S/BA) system was polymerized by means of a semicontinuous emulsion process, varying the composition of the comonomer feed to obtain forced composition copolymers (FCCs). Five different feeding profiles were used, searching for a scheme to obtain chains rich in S (looking for considerable modulus), and chains rich in BA (looking for large deformation) as a technique to achieve synergy in copolymer properties; the mechanostatic and dynamic characterization discloses the correspondence between WCD and the bulk properties. ^1^H-nuclear magnetic resonance (^1^H-NMR) analysis enabled the determination of the cumulative copolymer composition characterization, required to estimate the WCD. The static test (stress-strain) and dynamic mechanical analysis (DMA) were performed following normed procedures. This is the first report that shows very diverse mechanostatic performances of copolymers obtained using the same chemical system and global comonomer composition, forming a comprehensive failure envelope, even though the tests were carried out at the same temperature and cross head speed. The principles for synergic performance can be applied to controlled radical copolymerization, designing the composition variation in individual molecules along the conversion.

## 1. Introduction

Since the twentieth century, different blending methods, types of reactions, and polymerization processes have been used to prepare two component polymers, attempting to combine or optimize properties, expand applications, or simply optimize the cost/properties relationship [1,2,3]. It has been demonstrated that the contribution of each polymeric component is improved if phase separation occurs at the microscopic level, which implies that chemical blending offers better component interaction than physical blending [4]. Additionally, the mechanical superiority of polymer blends containing a gradient in composition over materials with uniform composition in the polymer bulk (e.g., gradient interpenetrating polymer networks (gIPNs) versus regular IPNs obtained by a three step method using bulk polymerization) has been shown [5,6,7]. Such superiority has been attributed to the synergic contribution of polymeric region(s) rich in component A as well as of region(s) rich in component B, in the spatial structure of the bulk polymer, which leads to the optimization of properties [6]. Moreover, using the suspension process, forming gradients by comers/monomer diffusion in polymer beads followed by an in situ polymerization, better control in transport properties [8,9], or ionic exchange optimization [10,11] has been achieved in polymer blends. Nevertheless, for materials obtained by conventional or traditional free radical polymerization (FRP), if sequential homopolymerizations of A and B components are used in emulsion, a two-phase core-shell type polymer is usually obtained, where phase separation generates a weak interaction between the components, inhibiting the optimization of properties. However, by appropriate comonomer mixing taking advantage of their common difference in reactivities [12], gradients in composition have been formed since a long time ago to produce for example a gradient in properties like refraction-index in copolymers [13]. Basically, gradients in polymers may be formed in one or more constituents of a polymeric material, carrying out variations in space, structure, composition, or synthesis method [14,15,16,17]. With that perspective, taking into consideration that covalent bonding offers the strongest component interaction and that gradients in composition help for the synergic contribution of the attributes of each component, the attainment of convenient gradual changes in the composition spectrum of copolymer chains (weight composition distribution of chains; WCD) could improve the mechanical properties. One way to search for these characteristics using free radical copolymerization (FRC) is to prepare copolymers by means of a semicontinuous process varying the feed comonomer composition along the reaction. That is, in principle forcing the reaction, copolymer chains rich in A, as well as chains rich in B can be formed, regardless of the relative reactivities (for not extremely distant values). Additionally, with a gradual variation in feeding composition, the formation of chains with intermediate compositions of monomers A and B can also be attained; such chains may serve as compatibilizers to reduce the tendency to separate the polymer bulk into two phases. With that aim, using the styrene-2-ethylhexyl acrylate system with a seeded multistage semicontinuous emulsion process, promising results were reported years ago using linear feeding profiles for the comonomers with variations in global composition [18]; besides, seed type or particle size may also be varied [19]. If the cumulative composition of the copolymer chains is followed along the reaction and used to estimate the WCD of the copolymer bulk, the copolymer properties can be correlated to that composition spectrum. With this aim, the styrene/*n-*butyl acrylate (S/BA) system with styrene seed was polymerized in emulsion, considering several feeding profiles for the 70/30 *w*/*w* composition. For the copolymers obtained, significant changes in the toughening effect caused by the BA contribution were observed, while their Young’s modulus was sustained to a certain extent at room temperature. Those changes in mechanical behavior corresponded to variations in the copolymer composition spectrum [20]. Based on those results, to demonstrate the full correspondence between the composition spectrum of the copolymer chains and the mechanical properties (dynamic and static) of a copolymer system, in this study several feeding profiles using the 50/50 *w*/*w* S/BA as chemical system and a semicontinuous emulsion process were considered and the correspondent WCDs are estimated. The S/BA monomer pair is a system of interest for the combination of rigidity-elasticity properties with distant glass transition temperatures (–55 °C for BA and 100 °C for S correspond to the homopolymers), and shows moderate phase segregation [21]. Their relative reactivities are 0.19 for BA and 0.72 for S [22].

For the reactions, starting with a polystyrene (PS) seed, predetermined feeding stages are used with linear, parabolic, or linear V type (e.g., linear descendant-ascendant) profiles to change the instantaneous S/BA comonomer composition and orientate, since at the beginning of the reaction the composition changes in the new copolymer chains. With the feeding schemes used, the formation of chains rich in S units (looking for a high modulus) and chains rich in BA units (for high deformation capacity) is intended. In the end, this approach may be used as a method to tailor the properties by means of useful gradual changes in the WCD of the copolymer chains. To be used as references for mechanical properties, an equivalent statistical copolymer (SC) and a core-shell type polymer in two stages (T-S) were also prepared and tested.

## 2. Experimental Section Materials

**Figure 1:**
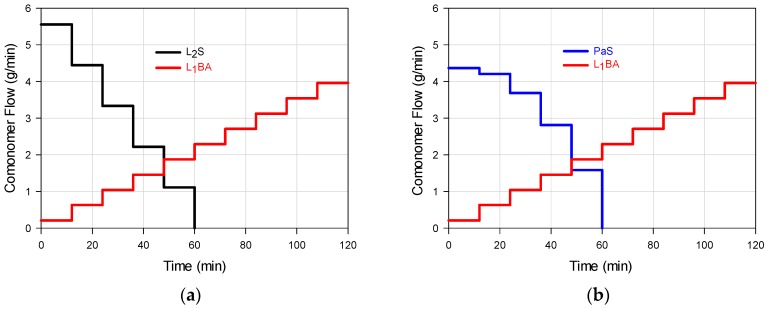

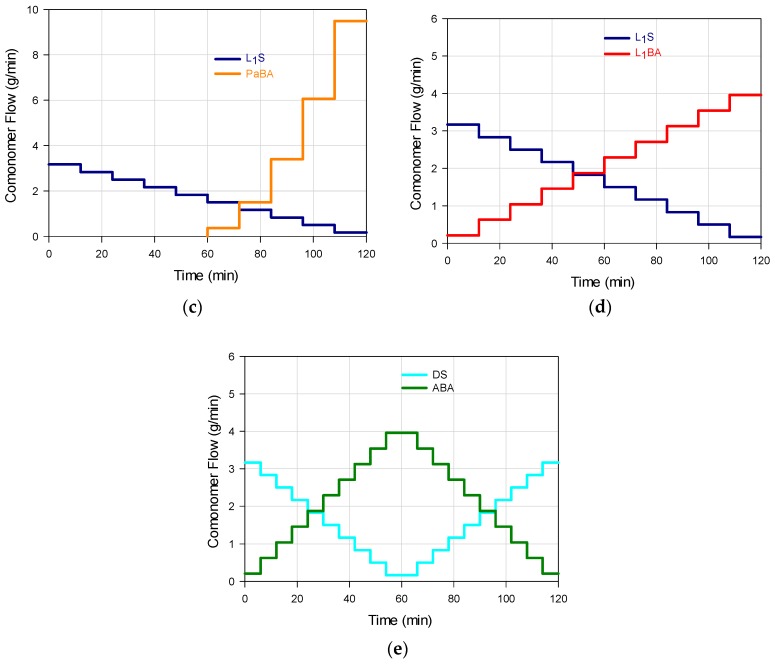
Comonomers flow as a function of reaction time, for S/BA FCCs synthesis: (**a**) M_1_ (L_2_S/L_1_BA); (**b**) M_2_ (PaS/L_1_BA); (**c**) M_3_ (L_1_S/PaBA); (**d**) M_4_ (L_1_S/L_1_BA); and (**e**) M_5_ (DS/ABA).

Styrene and *n*-butyl acrylate monomers (both from Sigma Aldrich, St, Louis, MO, USA, purity >99%) were disinhibited with ionic exchange resins; methyl ester hydroquinone resin was used for BA, and 4-tert-butylcathechol for S. As surfactant, sodium dodecylsulfate (SDS; Aldrich, St, Louis, MO, USA, purity >98%) was used as acquired. Potassium persulfate was used as initiator (KPS; Aldrich, purity >98%). Sodium bicarbonate (Arm and Hammer, Ewing, NJ, USA) was used to buffer pH changes during the reaction. Distilled water was used for every polymerization reaction. In all polymerizations, nitrogen gas was used to purge the reaction system. Twenty wt % of solid polymer (PS, T-S or copolymers) was obtained at the end of every reaction. The reactions were carried out at 70 °C in a 4 L glass reactor, stirring the system at 400 rpm; reaction times >12 h were used to maximize monomer(s) conversion (followed by gravimetry). The SC synthesis was carried out by adding quickly to the reactor the required amount of monomers (S/BA, 50/50 *w*/*w*) at room temperature while stirring. Before the addition of the required amount of initiator, the whole system was heated up to start the batch reaction. For the other polymeric materials, a PS seed was obtained first by batch emulsion polymerization. The T-S polymer was obtained by adding to the reactor the required amount of PS seed and for the second stage the required amount of BA monomer (BA/PS, 50/50 *w*/*w*) was quickly added to such a reactor while stirring; then, the whole system was heated up and a similar procedure to the one followed for the SC synthesis was carried out. The five forced composition copolymers (FCCs) were obtained by means of seeded semicontinuous emulsion processes; for the FCCs the global styrene content was also 50 wt %. At the start of each copolymerization reaction, the reactor was charged with a seed latex containing 50 g of PS and the amount of distilled water required to complete 1600 g of total load. Then, 10 sequential “comonomer feeding stages” of 12 min each one were implemented (except for the V type feeding profile where 20 stages of 6 min were used). At the beginning of each stage, an aqueous solution containing 45 g (except for the reaction of the V type feeding profile, where 22.5 g of water were used) of distilled water, and the required amounts of KPS, SDS, and NaHCO_3_ were added as a batch to the reaction system. In every stage, the amount added of each salt was the one corresponding to 2% of the total mass of the comonomers to be added in that stage. The feeding flow was modified at the start of each stage, and pumped to the reactor (Masterflex L/S 7528-10) at constant flow rate during the stage. Five feeding profile combinations for the S and BA comonomers were implemented (M_1_–M_5_), by means of linear (L_1_ or L_2_), parabolic (Pa), linear ascendant-linear descendant (A), and linear descendant-linear ascendant (D) trajectories. Such individual flow profiles are shown in Figure 1a–e with the following schemes: L_2_S/L_1_BA (M_1_); PaS/L_1_BA (M_2_); L_1_S/PaBA (M_3_); L_1_S/L_1_BA (M_4_); DS/ABA (M_5_); in all cases the feeding time was predetermined as 2 h.

**Figure 2:**
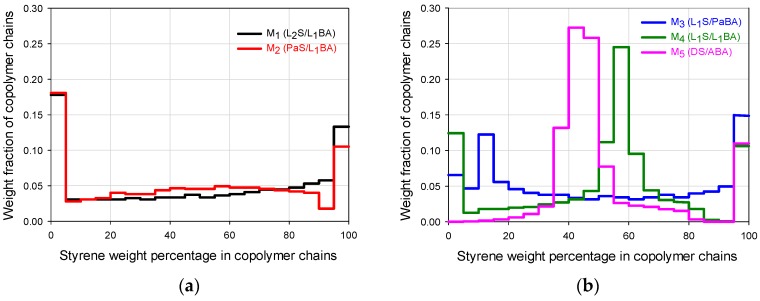
Weight fraction of copolymer chains of FCCs as a function of styrene weight percentage in the copolymer chains, estimated for: (**a**) M_1_, and M_2_, materials and (**b**) M_3_, M_4,_ and M_5_ materials. For codes, see Figure 1.

For the characterization of each FCC material, seven samples were extracted at predetermined reaction times, to be measured by ^1^H-NMR (Varian Gemini 2000, Agilent Technologies, Palo Alto, CA, USA) their respective global styrene percentage ($SP_{\exp}$ values) and, by gravimetry their respective global conversion values ($X_{\exp}$ values). For the calculations to build up the WCD, the conversion interval (from 0% up to the final experimental conversion value) was discretized in subintervals of 0.1% in width. It was considered that all the chains produced inside of each conversion subinterval *i* (characterized by its respective $X_{i}$ value) have the same $SP_{i}$ value, which was estimated with a third order polynomial obtained by fitting the seven above mentioned experimental values; for this, the least squares method was used. The total mass of monomer *j* (S or BA) polymerized up to subinterval *i* ($m_{\mathrm{up}\;\mathrm{to}\;i}^{j}$) can be calculated with the following equations: $m_{\mathrm{up}\;\mathrm{to}\;i}^{S}=X_{i}M_{T}SP_{i}$ or, $m_{\mathrm{up}\;\mathrm{to}\;i}^{\mathrm{BA}}=X_{i}M_{T}\left(100-CP_{i}\right)$, $M_{T}$ being the total amount of monomer (S plus BA) included in the reaction (450 g). Thus, the mass of monomer *j* polymerized in the subinterval *i* $\left(\Delta m_{i}^{j}\right)$ can be estimated as follows: $\Delta m_{i}^{j}=\left(m_{\mathrm{up}\;\mathrm{to}\;i+1}^{j}-m_{\mathrm{up}\;\mathrm{to}\;i}^{j}\right)$. Therefore, the mass percentage of S in the chains formed in the subinterval *i* $\left(\%S_{i}\right)$ can be obtained with the following equation: $\%S_{i}=\left(\Delta m_{i}^{S}\right)/\left(\Delta m_{i}^{S}+\Delta m_{i}^{\mathrm{BA}}\right)$. The respective masses of polymer produced with $\%S_{i}$ values within the pre-established composition intervals (e.g., 0%–5% of S, 5%–10% of S and so on) were suitably summed up and, with such values, the histograms showing the WCD profiles for the copolymer chains contained in each FCC material at the end of the reaction (Figure 2a,b) were constructed [19,20].

All latexes were dried by evaporation at room temperature; the collected high molecular weight polymers (such as the ones reported before [18,19,20]), were used to prepare samples by compression molding (Schwabenthan polystat 200T) to carry out all mechanical tests. Stress-strain measurements were performed at 25 °C following the procedure of the American Society for Testing Materials (ASTM D638, samples Type IV, crosshead speed: 0.0083 cm/s; Universal testing machine SFM10).

A dynamic mechanical analysis (DMA) was carried out to measure storage and loss moduli as a function of temperature (ASTM D-5023, three point bending clamp, 1 Hz, heating rate: 1 °C/min TA Instruments Q800, Waters Corporation, New Castle, DE, USA).

## 3. Results and Discussion

**Figure 3:**
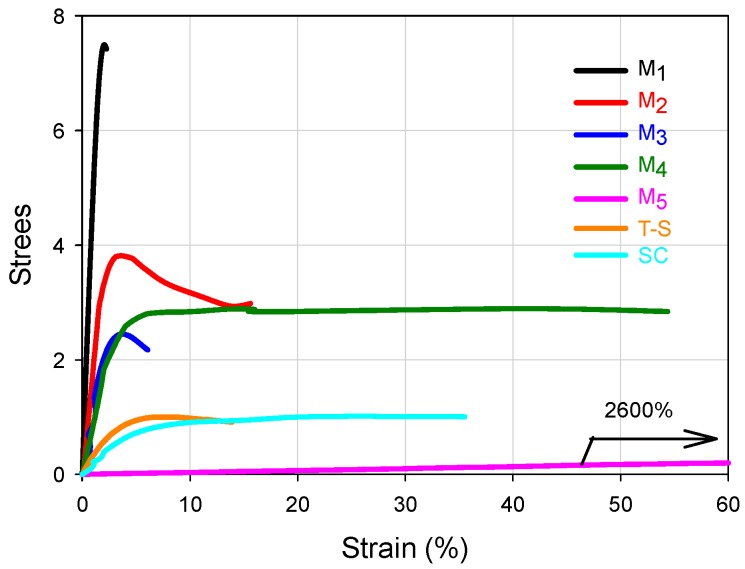
Stress-strain behavior of FCCs (M_1_–M_5_), two stage polymer (T-S) and statistical copolymer (SC) at 25 °C (crosshead speed: 0.5 cm/min). For FCCs codes, see Figure 1.

**Figure 4:**
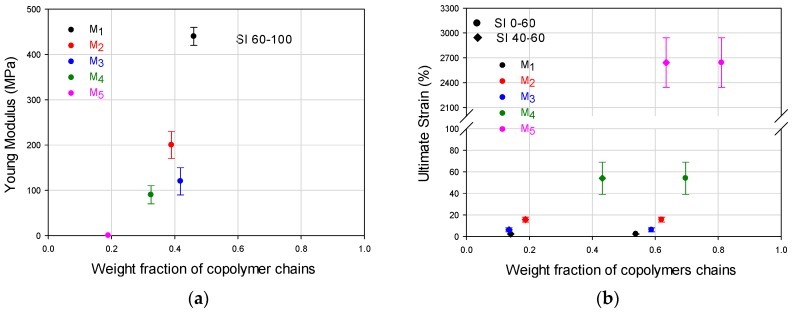
Young’s modulus and ultimate strain as a function of styrene weight fraction within FCCs at specific copolymer composition intervals. (**a**) Young modulus for SI 60–100; (**b**) Ultimate strain for SI 0–60 and SI 40–60. For FCCs codes, see Figure 1.

**Figure 5:**
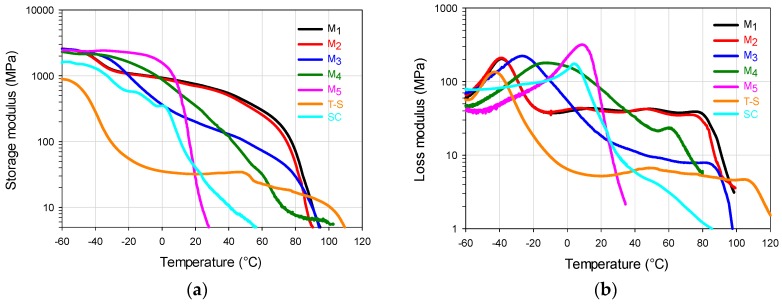
(**a**) Storage modulus; and (**b**) loss modulus as a function of temperature for: FCCs (M_1_–M_5_), T-S and SC materials (frequency: 1 Hz). For codes, see Figure 1 and Figure 2.

The chosen feeding profiles (Figure 1a–e) allow the diversification of composition histograms that represent the WCDs of the FCCs presented in Figure 2a,b. The feed trajectories allow the formation of chains that are rich in styrene or butyl acrylate at different moments depending on the flow profile and the monomer reactivities; the continuous change in monomer composition allows a biased change in the instantaneous copolymer composition. The composition profiles shown in Figure 2a,b define the mechanical properties that are presented in Figure 3, Figure 4 and Figure 5. Even though the copolymer composition determines the mechanical performance that may vary from highly elastic (almost pure poly(butyl acrylate) (PBA), to highly rigid behavior (almost pure PS), the deformation capacity and moduli are affected by both components depending on the chains buildup along the composition spectrum. The correlation between the WCD of a copolymer and its correspondent mechanical properties can be disclosed, quantifying in Figure 2a,b the weight fraction accumulation or wt % of the copolymer chains within specific composition ranges (e.g., 0–60 wt %, which can be designated as styrene composition interval or simply SI 0–60).

For the general FCC behavior, even though the chains that are close to an extreme of the ordinate axis will have significant influence on the property related to the pure homopolymer (i.e., at 0 wt % the behavior stands for PBA and at 100 wt % to PS), the synergic performance also requires the considerable presence of chains that contain both components (as a fundamental difference to T-S materials). In that sense, looking at the FCC histograms as a whole in Figure 2a,b, the highest mechanical moduli can be expected for M_1_ and M_2_, but higher deformations can be expected for M_4_ or M_5_.

For the FCCs presented in Figure 3, it can be seen that the set of stress-strain curves at 25 °C encompass an extensive range of mechanical performance, forming in fact a wide failure envelope (although it was obtained at the same temperature and cross-head speed) [23], which only depends on the WCD of such materials, because they possess the same global composition. The Young’s modulus (*E*) and deformation capacity ranks for the FCCs can be seen in Table 1 and their correlations with the WCD can be established by observing Table 2. In principle, the higher the modulus, the lower the deformation; that is, basically the FCCs occupy opposite position values in E and deformation ranks. However, looking at the curves of Figure 3, it can be inferred that the design of the WCDs may be used to obtain a good combination of *E* and deformation, searching for considerable rigidity and toughness.

In Figure 3, M_1_ and M_5_ clearly show the highest and lowest modulus respectively whose values can be seen in Table 1. In Table 2, it can be noticed that they present significant differences in favor of M_1_ for the wt % of copolymer chains that are rich in S (SI 70 or 80–100), and even since SI 60–100, because the wt % content of copolymer chains for that range are 46.1 for M_1_ versus 18.8 for M_5_. In contrast, for the composition ranges below 60 wt % S, the big differences are in favor of M_5_ especially for the SI 40–50 region (53.3 wt % for M_5_ versus 7.1 wt % of chains for M_1_), or the accumulated for SI 0–60 (53.9 for M_1_ versus 81.2 for M_5_), denoting the high capacity of M_5_ for deformation. Such extreme stress-strain behaviors may be used as a basis to look at the general trends for the FCCs.

Comparing the differences for the M_2_–M_4_ materials regarding Young modulus, in Figure 3 the curves almost overlap at the origin and that proximity is reflected in Table 1 considering the standard deviation of the experimental values. Considering the general mechanical behavior the contribution of the chains accumulated in SI 60–100 for the modulus and SI 0–60 for the deformation capacity, those mechanical properties for M_1_–M_5_ materials are plotted in Figure 4a,b, to show their trends with weight fraction of copolymer chains. In addition, since M_4_ and M_5_ show high weight fraction values in SI 40–60, their deformation values are also plotted for that interval (Figure 4b). In Figure 4a, the positive slope shows how the modulus increases as the weight fraction increases in the SI 60–100 interval. It can also be seen that the modulus difference is not very significant for M_2,_ M_3_ and M_4_; however, looking at the ultimate strain in Figure 4b and Table 1, M_2_ and M_3_ show values close to each other, while M_4_ is considerable bigger than both of them (M_4_ shows 3.4 and 8.8 times the modulus value of M_2_ and M_3_ respectively). This behavior clearly shows the synergic performance of M_4_, which in fact can also be perceived in Figure 3 looking at M_2_–M_4_ curves, and comparing the toughness in Table 1, where M_4_ shows 3.1 and 12.8 times the value of M_2_ and M_3_ respectively. The positive slope for the deformation trend of the FCCs in Figure 4b, that can be observed for both composition intervals (SI 0–60 and SI 40–60), denotes the importance of the presence of copolymer chains with intermediate compositions.

Looking at the reference materials in Figure 3, the T-S polymer shows a low modulus compared to four FCCs and similar deformation capacity to M_2_, while the SC shows almost three times its deformation capacity, although it has a lower modulus than the T-S material. Such performances validate the convenience to synthesize copolymers by the procedure proposed here and confirm that in the T-S polymeric materials, the properties combination of the components is not promoted due to phase separation of the homopolymers within the polymer bulk.

In Figure 5a, for the storage modulus (*E*’) of the two-component polymers it can be seen that the high value in SI 0–60 of the M_5_ copolymer promotes the *E*’ decay at low temperature, while the high values in SI 60–100 and SI 80–100 of M_1_ impart the highest temperature resistance of the FCCs. The M_2_ material follows closely the M_1_ trajectory up to 40 °C with an *E*’ decrease at slightly lower temperature due to its lower value for the SI 60–100, but mainly to its lower value in SI 80–100. The *E*’ decay that M_1_ and M_2_ materials show at PBA *T*_g_ essentially correspond to the high value in SI 0–20, that both show in Figure 2a and Table 2. For the SI 0–30, the M_3_ copolymer that shows the highest value in Table 2 presents a steeper *E*’ decay that starts slightly above PBA *T*_g_, but its high value in SI 80–100 allows a thermal resistance before the catastrophic E’ final decay at 80 °C.

The M_4_ copolymer does not show a phase separation tendency in *E*’, starting a smooth decay at 0 °C and, around 40 °C it presents a crossover point with the M_3_ curve, denoting a slightly higher temperature resistance for the latter material, when both approach the terminal zone for *E*’. The SC shows very poor temperature resistance for the *E*’ value, while the T-S shows a marked decay at PBA *T*_g_ (more than one order of magnitude) followed by a plateau that starts at −20 °C that presents a final decay close to 100 °C. Its low *E*’ performance as in the static test can be attributed to the lack of interaction between components.

For loss modulus (*E*”) in Figure 5b, the M_5_ FCC shows a copolymer type behavior with a peak at approximately 10 °C with an almost vertical decay afterwards, denoting an elastomeric behavior at room temperature. M_1_ and M_2_ copolymers follow very similar trajectories (as in *E*’ behavior) in the whole test temperature range. The peak around −40 °C is assigned to a glass transition response, due to the chains accumulated in the SI 0–20 (Figure 2a); then, *E*” maintains a certain plateau value, until it falls at 80 °C, in accordance with *E*’ decay (Figure 5a). M_3_ material, shows a wide peak at −25 °C, because there are a considerable amount of chains in the SI 0–20; then, it shows a smooth decay as it did in the *E*’ curve (Figure 5a). At around 40 °C, it starts a plateau like behavior due to the influence of the SI 60–100, until its final decay at approximately 80 °C.

The M_4_ material shows a very wide peak from −40 to 40 °C (the widest of all polymeric materials) indicating the presence of copolymer chains with a wide range of compositions around the highest peak value (approximately at −11 °C); that trajectory is highly influenced by the chains composition profile in the SI 0–70 range. The slow decrease of E” that starts at ca. 0 °C continuous until it presents a shoulder formation tendency at 60 °C, which is a signal influenced by the accumulation of chains in the SI 70–100 range (chains rich in S); the final modulus decay occurs at lower temperature than M_1_–M_3_ materials due to its lower SI 70–100 value. The high *E*” value from −55 °C to almost 40 °C, indicates a high energy dissipation capacity (area under the curve) within that temperature range. For the reference materials, the SC presents a copolymer type behavior with a lower but slightly wider peak than the one presented by M_5_ (accounting for the dissipation capacity since −60 °C), that deviates slightly at 20 °C towards its final decay, denoting the random structure with very low temperature resistance. The T-S material shows a peak at −45 °C showing a temperature peak value close to PBA *T*_g_ with a significant *E*” decay (due to is high PBA content) that is followed by a plateau from 0 to ca. 100 °C (showing there a small peak), denoting the glass transition temperature values that resemble the ones of the pure homopolymers.

In summary, the methodology proposed here to design the properties of a FCC by variations in the WCD of the chains and its correlation with the mechanical properties has been demonstrated, showing also an unusual failure envelope involving diverse mechanostatic performances of copolymers obtained using the same chemical system and global comonomer composition, tested at the same temperature and cross head speed. Additionally, as stated in this work, the correlation between WCD profiles and the mechanical properties above described using FRP reactions (establishing the structure characteristics required for synergic behavior) has an equivalent with controlled radical polymerization (CRP). Recently, for the S/BA system using also a semicontinuous emulsion process with a multistage feeding technique [18,19,20]), Guo et al. tested two types of gradient copolymers obtained by CRP that exhibited yielding and elastic deformation [24], whose copolymer chains composition varied from one component to the other, according to their former report [25]. Although they did not present a discussion about their behavior related to the specific structures of those gradient copolymers, the explanation for that behavior is in accordance with the synergic contribution of the components proven here (as it was also demonstrated in chemical blends); the presence of long segments of each one of the components along with transition chain segments of intermediate compositions of monomers A and B in between each molecule, should lead to the optimization of polymer properties. The conformed polymer bulk would be similar to the copolymers above described using FRC. In another report, similar results on damping performance were found for materials obtained by FRC [20] and CRP [21] for the S/BA system. The equivalence in properties has to do with the richness in S and BA units. For the FRC case, such richness occurs in different chains, and for the CRP case it occurs within one molecule. Such component richness in high MW chains is important for mechanical properties in both cases, because the contribution of each component is enhanced in that way; it is well known that MW is an important parameter for mechanical properties [26,27]. Additionally, the polydispersity index should also be considered if looking for uniform properties in the product [28], or for easy processing, particularly for high MW polymers [29]. To improve the synthesis processes, the use of programmed feeding can help to control comonomer addition in FRC [30] or CRP [31,32].

## 4. Conclusions

The WCD-bulk mechanical properties relationship for FCCs obtained by FRP has been demonstrated. Additionally, it has been shown that the WCD can be easily modified by variations in the comonomer feeding profile if a semicontinuous emulsion polymerization is used (taking into consideration the specific monomer reactivities). The wide failure envelope obtained confirms that the methodology presented here can be used to tailor copolymer properties using a reaction system that can easily be extended to industrial scale for a wide variety of applications.

## Figures and Tables

**Table 1:** Young’s modulus and ultimate properties of FCCs (M_1_–M_5_), as well as T-S and SC materials at 25 °C and crosshead speed of 0.5 cm/min. For codes, see Figure 1 and Figure 2.

Property	M_1_	M_2_	M_3_	M_4_	M_5_	T-S	SC
Young Modulus (MPa)	440 ± 20	200 ± 30	120 ± 30	90 ± 20	0.35 ± 0.10	30 ± 30	20 ± 4
Ultimate Stress (MPa)	7.4 ± 0.8	3.0 ± 0.4	2.2 ± 0.3	2.8 ± 0.4	0.12 ± 0.07	0.9 ± 0.1	1.0 ± 0.1
Ultimate Strain (%)	2.2 ± 0.2	15.6 ± 2.3	6.1 ± 1.8	54 ± 15	2600 ± 300	13.9 ± 1.9	35.5 ± 1.1
Toughness (MPa)	11.0 ± 0.6	48.5 ± 0.9	11.7 ± 1.0	150 ± 2	630 ± 5	11.7 ± 0.7	31.5 ± 2.4

**Table 2:** Weight fraction of copolymer chains of FCC samples whose composition is within a specific interval. For codes, see Figure 1.

Styrene wt % in Cop. Chains	M_1_	M_2_	M_3_	M_4_	M_5_
0–10	0.209	0.207	0.112	0.134	0.001
10–20	0.062	0.063	0.177	0.035	0.005
20–30	0.063	0.078	0.086	0.040	0.017
30–40	0.064	0.081	0.075	0.050	0.153
40–50	0.071	0.091	0.064	0.073	0.533
50–60	0.070	0.094	0.070	0.349	0.103
60–70	0.079	0.094	0.065	0.137	0.044
70–80	0.090	0.089	0.072	0.056	0.033
80–90	0.101	0.081	0.081	0.020	0.000
90–100	0.191	0.122	0.197	0.105	0.110
0–20	0.271	0.270	0.289	0.169	0.006
0–30	0.334	0.348	0.375	0.209	0.023
0–60	0.539	0.614	0.585	0.682	0.812
30–60	0.205	0.267	0.209	0.473	0.789
40–60	0.141	0.186	0.134	0.422	0.636
60–80	0.169	0.183	0.137	0.193	0.077
60–100	0.461	0.386	0.415	0.318	0.188
70–100	0.382	0.292	0.350	0.182	0.143
80–100	0.292	0.203	0.278	0.125	0.110
0–100	1.000	1.000	1.000	1.000	1.000

## References

[1] Allcock H.R., Lampe F.W. (1990). Contemporary Polymer Chemistry.

[2] Gum W.F., Riese W., Ulrich H. (1992). Reaction Polymers: Chemistry, Technology, Applications, Markets.

[3] Matyjaszewski K. (1998). Controlled Radical Polymerization.

[4] Manson J.A., Sperling L.H. (1976). Rubber-Toughened Plastics in Polymer Blends and Composites.

[5] Jasso C.F., Hong S.D., Shen M., Cooper S.L., Estes G.M. (1979). Stress-Strain Behavior of PMMA/ClEA Gradient Polymers in Multiphase Polymers.

[6] Jasso-Gastinel C.F., Salamone J.C. (1996). Gradient Polymers in Polymeric Materials Encyclopedia.

[7] Karabanova L.V., Mikhalovsky S.V., Lloyd A.W., Boiteux G., Sergeeva L.M., Novikova T.M., Lutsyk E.D., Meikle S. (2005). Gradient semi-interpenetrating polymer networks based on polyurethane and poly(vinyl pyrrolidone). J. Mater. Chem..

[8] Mueller K.F., Heiber S.J. (1982). Gradient-IPN-modified hydrogel beads: their synthesis by diffusion-polycondensation and function as controlled drug delivery agents. J. Appl. Polym. Sci..

[9] Mueller K.F., Heiber S.J. (1983). Membrane Modified Hydrogels. U.S. Patent.

[10] Jasso-Gastinel C.F., Garcia-Enríquez S., Gonzalez-Ortiz L.J. (2008). Synthesis and characterization of anionic exchange resins with a gradient in polymer composition for the PS-*co*-DVB/PDEAMA-*co*-DVB system. Polym. Bull..

[11] Jasso-Gastinel C.F., González-Ortiz L.J., García-Enríquez S. (2013). Proceso para la Síntesis de Resinas de Intercambio Iónico que Presenten un Gradiente Continuo de Composición en la Sección Iónica de la Partícula, Mediante Difusión Monomérica. MX Patente.

[12] Odian G. (2004). Chain Copolymerization in Principles of Polymerization.

[13] Ohtsuka Y. (1973). Light-focusing plastic rod prepared form diallyl isophthalate-methyl methacrylate copolymerization. Appl. Phys. Lett..

[14] Bever M.B., Duwez P.E. (1972). Gradients in composite materials. Mater. Sci. Eng..

[15] Shen M., Bever M.B. (1972). Gradients in polymeric materials. J. Mater. Sci..

[16] Beginn U. (2008). Gradient copolymers. Colloid Polym. Sci..

[17] Jasso-Gastinel C.F. (2016). Gradients in Polymers in Encyclopedia of Biomedical Polymers and Polymeric Biomaterials.

[18] Jasso C.F., Reyes I., Lopez L.C., González L.J. (2006). Mechanical performance of styrene-2-ethylhexyl acrylate polymers synthesized by semicontinuous emulsion polymerization varying feed composition. Int. J. Polym. Anal. Charact..

[19] Núñez-Pérez F.A. (2012). Efecto de Diversos Parámetros de Composición y Morfología en las Propiedades Mecánicas de un Sistema en Emulsión. Sistema: Acrilato de Butilo/Estireno. Ph.D. Thesis.

[20] Arnez-Prado A.H., González-Ortiz L.J., Aranda-García F.J., Jasso-Gastinel C.F. (2012). The variation of comonomers feeding profile to design the distribution of chains composition for the optimization of the mechanical properties in copolymer systems. e-Polymers.

[21] Mok M.M., Kim J., Torkelson J.M. (2008). Gradient copolymers with broad glass transition temperature regions: Design of purely interphase compositions for damping applications. Polym. Phys..

[22] Kostanski L.K., Hamielec A.E. (1992). Influence of temperature on butyl acrylate—Styrene copolymerization parameters. Polymer.

[23] Rodriguez F., Cohen C., Ober C.K., Archer L.A. (2015). Ultimate Properties in Principles of Polymer Systems.

[24] Guo Y., Gao X., Luo Y. (2015). Mechanical properties of gradient copolymers of styrene and *n-*butyl acrylate. J. Polym. Sci..

[25] Guo Y.L., Zhang J.H., Xie P.L., Gao X., Luo Y.W. (2014). Tailor-made compositional gradient copolymer by a many-shot RAFT emulsion polymerization method. Polym. Chem..

[26] Nielsen L.E., Landel R.F. (1994). Dynamical Mechanical Properties in Mechanical Properties of Polymers and Composites.

[27] Nielsen L.E., Landel R.F. (1994). Stress-Strain Behavior and Strength in Mechanical Properties of Polymers and Composites.

[28] Elsen A.M., Li Y., Li Q., Sheiko S.S., Matyjaszewsky K. (2014). Exploring quality in gradient copolymers. Macromol. Rapid Commun..

[29] Clegg P.L., Ogorkiewicz R.M. (1969). Introduction and Basic Considerations in Thermoplastics: Effects of Processing.

[30] Yang W., Xie D., Seng X., Zhang X. (2013). Sequence distribution and cumulative composition of gradient latex particles synthetized by a power-feed technique. Ind. Eng. Chem. Res..

[31] Sun X., Luo Y., Wang R., Li B.G., Zhu S. (2008). Semibatch RAFT polymerization for producing ST/BA copolymers with controlled gradient composition profiles. AIChE J..

[32] Li X., Mastan E., Wang W.J., Li B.G., Zhu S. (2016). Progress in Reactor Engineering of controlled radical polymerization: a comprehensive review. React. Chem. Eng..

